# Short term Heart Rate Variability to predict blood pressure drops due to standing: a pilot study

**DOI:** 10.1186/1472-6947-15-S3-S2

**Published:** 2015-09-04

**Authors:** G Sannino, P Melillo, S Stranges, G De Pietro, L Pecchia

**Affiliations:** 1 Institute of High-Performance Computing and Networking (ICAR) of the National Research Council of Italy (CNR), Naples, 80131, Italy; 2 Multidisciplinary Department of Medical, Surgical and Dental Sciences, Second University of Naples, Naples, 80138, Italy; 3 Department of Population Health, Luxembourg Institute of Health (LIH), 1A-B, rue Thomas Edison, L-1445 Strassen, Luxembourg; 4 School of Engineering, University of Warwick, CV47AL, UK

**Keywords:** Heart Rate Variability, standing hypotension, fall prediction in the elderly

## Abstract

**Background:**

Standing from a bed or chair may cause a significant lowering of blood pressure (ΔBP), which may have severe consequences such as, for example, falls in older subjects. The goal of this study was to develop a mathematical model to predict the ΔBP due to standing in healthy subjects, based on their Heart Rate Variability, recorded in the 5 minutes before standing.

**Methods:**

Heart Rate Variability was extracted from an electrocardiogram, recorded from 10 healthy subjects during the 5 minutes before standing. The blood pressure value was measured before and after rising. A mathematical model aiming to predict ΔBP based on Heart Rate Variability measurements was developed using a robust multi-linear regression and was validated with the leave-one-subject-out cross-validation technique.

**Results:**

The model predicted correctly the ΔBP in 80% of experiments, with an error below the measurement error of sphygmomanometer digital devices (±4.5 mmHg), a false negative rate of 7.5% and a false positive rate of 10%. The magnitude of the ΔBP was associated with a depressed and less chaotic Heart Rate Variability pattern.

**Conclusions:**

The present study showes that blood pressure lowering due to standing can be predicted by monitoring the Heart Rate Variability in the 5 minutes before standing.

## Background

An orthostatic change, namely a change in body posture from sitting to standing, causes specific changes in heart rate and blood pressure as a compensatory reaction of the body. In fact, in the few minutes after standing, there is a redistribution of the blood volume and a pooling of blood in the lower extremities due to gravitational forces. As a consequence, the venous return to the heart falls and the cardiac filling pressure is reduced diminishing the stroke volume and cardiac output. Therefore, there is a drop in blood pressure (ΔBP) due to the change of position, a condition referred to as Standing Hypotension (SH).

To avoid dizziness or fainting due to the limited blood supply to the brain, the blood pressure in the large arteries decreases to compensate and regulate. The objective of this regulatory mechanism is to achieve normal blood pressure as quickly as possible while providing an adequate supply of blood to the vital organs.

Healthy subjects respond with an autonomic adjustment, which increases vascular tone, heart rate and cardiac contractility, and stabilizes arterial pressure [1]. In particular, the sympathetic outflow to the heart and blood vessels increases and the cardiac vagus nerve activity decreases. In healthy subjects, during standing, the contraction of the lower body skeletal muscles prevents excessive pooling and augments the venous return to the heart.

Therefore, the dynamic of the blood pressure and particularly the capability to restore homeostasis after standing is strongly dependent on the status of the Autonomous Nervous System (ANS). Starting from the hypothesis that the magnitude of the ΔBP a few minutes after standing is dependent on the status of the ANS before standing, which can be estimated using short term Heart Rate Variability (HRV), the research question of the study was: is it possible to predict ΔBP using the HRV measurements recorded in the 5 minutes before standing?

The prediction of this ΔBP is particularly relevant as at least 30% of the indoor falls of older subjects happen when they are rising from a bed or chair, such falls in the majority of the cases being due to the ΔBP caused by the activity of standing. Therefore, a model that could predict this ΔBP could help to predict a significant percentage of these falls.

The current paper proposes a mathematical model to predict the ΔBP due to standing in healthy subjects, based on the HRV features extracted from an ECG recorded, through the use of wearable sensors, in the 5 minutes before standing.

## Methods

### Study population and ethical approval

This study was conducted on a group of 10 healthy subjects, enrolled in accordance with the following selection criteria, namely that they were:

• no suffering from any pathological cardiovascular conditions, neurological or psychiatric disorders [2,3] or other severe diseases;

• not taking any medication at the time of the study;

• not professional athletes or high-level sport participants;

• had not taken any caffeine or alcohol in the 12 hours prior to the measurements.

The Biomedical and Scientific Research Ethics Committee (BSREC) of the University of Warwick approved the experimental protocol and each subject was given detailed information about the study and gave his/her informed consent before starting the measurements.

### Experimental protocol

The protocol was defined to maximize the repeatability and reproducibility of the experiments and aimed to simulate as far as possible the real life action of standing from a bed.

During the tests, an ECG signal was monitored using a one-lead wearable electrocardiogram sensor, the BH3-M1 (Zhephyr Ltd), attached to lightweight patches (33 grams) using standard ECG electrodes. In addition, a 3 leads ECG was recorded using a wearable biomedical amplifier, the Nexus 10 (MindMedia Ltd) in order to benchmark the signals acquired with the BH3-M1.

The BP was measured with a digital sphygmomanometer, the M2 basic (OMRON Ltd), with the left arm comfortably positioned on a horizontal surface and the cuff positioned at the level of the heart at about 2 cm from the inner side of the elbow. The experiments were carried out in a quiet room, with dimmed lighting and a comfortable temperature of about 23°C. All the experiments were carried out at the same time of the morning to minimize the circadian effects on the HRV [4].

**Figure 1:**
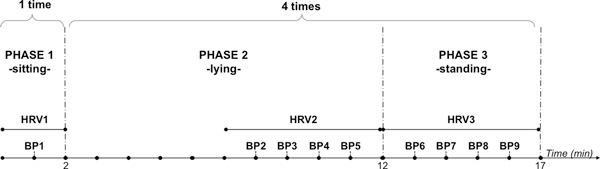
**Experimental Protocol**.

The protocol was composed of three phases (sitting, lying and standing) as described below (see Figure 1):

• Phase 1 (Sitting): the volunteers were invited to sit in a comfortable position for a baseline recording of BP and an ECG (>2 minutes).

• Phase 2 (Lying): the volunteers were invited to lie down in a supine position at about 45 cm from the ground for 10 consecutive minutes (5 minutes of resting + 5 minutes for the recordings): systolic and diastolic BP was recorded four times (with a 60 second interval) in the final 5 minutes before standing; an ECG was recorded during these final 5 minutes.

• Phase 3 (Standing): the volunteers were invited to stand up actively (without any help) and to stay in an upright position for 5 minutes. The subjects were all trained to stand up in a uniform manner: tilting the trunk and simultaneous twisting the body to the left; putting on the floor first the left and then the right foot; resting for five seconds; and finally standing up without using their hands to help. Once standing, systolic and diastolic BP was recorded four times (with a 60 second interval) and an ECG was recorded continuously for 5 minutes.

After standing the subjects were asked to report any symptoms of vertigo or dizziness and, if this was present, its magnitude (moderate or severe). Phases 2 and 3 were repeated four times after 5 minutes of resting. The whole protocol lasted approximately 100 minutes for each subject.

### Signal registrations

During phases 2 and 3, HRV excerpts of 5 minutes length were extracted from the ECG recordings (Figure 1).

### Signal feature extraction

The ECG measurements were pre-processed by using Kubios [5,6], a Matlab based software package for event-related bio-signal analysis developed by the University of Kuopio, Finland. Kubios is an advanced computer program to extract and analyse HRV.

Each ECG record is cleaned from power line interference, and muscle and movement artefacts, by using a cubic spline interpolation method [7,8]. The distance between two successive heart beats in a normal rhythm determines the RR interval, also called the NN interval. Since artefacts in the RR interval (i.e. the intervals between consecutive R peaks in the ECG signals) time series may interfere with the analysis of these signals, we adopted the Kubios Artefact removal RR filter with a maximum threshold of 5%.

The filtered signals were then analysed. A standard linear HRV analysis was performed according to the guidelines of the European Society of Cardiology and the North American Society of Pacing and Electrophysiology [9]. Additionally, non-linear features were computed according to the literature [10,11]. All the computed measurements are summarized in Table 1. Further details about the methodological conventions adopted are described in the three open access papers [12-14]

**Table 1:** Linear and Non-Linear Heart Rate Variability measurements selected in the current study.

	*Measure*	*UNIT*	*Description*
**Time-Domain**			

1	MeanRR	ms	Mean of RR intervals

2	STDNN	ms	Standard deviation of RR (NN) intervals

3	RMSSD	ms	Square root of the mean squared differences between successive RR intervals

4	NN50	count	Number of successive RR interval pairs that differ by more than 50 m

5	pNN50	%	NN50 divided by the total number of RR intervals

6	HRVtri		Integral of the RR interval histogram divided by the height of the histogram

7	TINN	Ms	Baseline width of the RR interval histogram

**Frequency-Domain**			

8	LF	ms2	Absolute powers of LF band, calculated with AR

9	HF	ms2	Absolute powers of HF band, calculated with AR

10	LF/HF		Ratio between LF and HF band powers

**Nonlinear-Domain**			

11	SD1	ms	Standard deviation of the Poincar´e plot perpendicular to the line of identity

12	SD2	ms	Standard deviation of the Poincar´e plot along the line of identity

13	ApEn		Approximate entropy

14	SampEn		Sample entropy

15	D2		Correlation Dimension

16	DFA1		Short-term fluctuation slope in Detrended Fluctuation Analysis

17	DFA2		Long-term fluctuation slope in Detrended Fluctuation Analysis

18	RPLmean	beats	Recurrent Plot (RP) mean line length

19	RPLmax	beats	RP maximum line length

20	RPREC	%	RP Recurrence Rate

21	RPDET	%	RP Determinism

22	RPShan		RP Shannon Entropy

### Raw Dataset

The HRV measurements were associated with the BP measurements as described in Figure 1 and collected in a database. According to the protocol, the following data were recorded for each subject:

• 1 HRV excerpt of 2 minutes, 1 systolic and 1 diastolic BP measurement during phase 1 (sitting).

• 1 HRV excerpt of 5 minutes, 4 systolic and 4 diastolic BP measurements during phase 2 (lying), which were repeated four times.

• 1 HRV excerpt of 5 minutes, 4 systolic and 4 diastolic BP measurements during phase 3 (standing), which were repeated four times.

Therefore, the database contained nine instances for each subject. Each instance *i *in the database was constituted by the following 30 pieces of information:

• sub_id: a number value from 1 to 10 to univocally identify each subject;

• HRV_id: a number value indicating one of the three HRV excerpts related respectively to the three phases (phase 1: sitting, phase 2: lying or phase 3:standing);

• test_id: a number value from 1 to 4 indicating the experiment repetition;

• symptom_id: a number value form 0 to 2 to indicate if the subject feels any manifestation of vertigo/dizziness: 0 = no vertigo; 1 = moderate vertigo; 2 = major vertigo;

• SYS_BP*_i_*: the 9 values of the Systolic Blood pressure measured;

• DIA_BP*_i_*: the 9 values of the Diastolic Blood pressure measured;

• *f*: a vector containing the 22 HRV measurements reported in Table 1;Therefore, each instance "*i*" was defined as follows:

(1) $$i=sub_{id}\;;\;\text{HR}\;{\text{V}}_{\text{id}}\;;\;\text{tes}{\text{t}}_{\text{id}}\;;\;\text{sympto}{\text{m}}_{\text{id}}\;;\;\text{SY}{\text{S}}_{\text{BPi}}\;;\;\text{DI}{\text{A}}_{\text{BPi}}\;;\;\bar{\text{f}}$$

### Predictive dataset

In order to develop and test a model to predict the magnitude of the BP drop, a second dataset was generated. The new dataset, hereinafter referred to as the *predictive dataset*, contained for each subject 4 instances defined as follows:

(2) $$i=sub_{id}\;;\;HRV_{id}=\text{2}\;\;;\;test_{id}\;;\;symptom_{id}\;;\;\text{Δ}SYS_{BP}\;;\;\text{Δ}DIA_{BP}\;;\;{\overset{¯}{f}|}_{\;phaseid=2}$$

where:

(3) $$\Delta SYS_{BP}=\left(SYS_{BP7}-\frac{\sum_{I=1}^{4}SYS_{BPi}{|}_{HRV_{ID}=2}}{4}\right)=BP7-mean(BP2:BP5)$$

and

(4) $$\Delta DIA_{BP}=\left(DIA_{BP7}-\frac{\sum_{I=1}^{4}DIA_{BPi}|{}_{HRV_{ID}=2}}{4}\right)=BP7-mean(BP2:BP5)$$

In other words, the predictive database contained the HRV measurements extracted from the 5 minute ECGs recorded in phase 2 (while lying) associated with the drop of systolic and diastolic BP detected in phase 3 (after standing). The BP drop (ΔBP) was calculated as the difference between the BP value measured during the 2^nd ^minute after the standing action (BP7) and the mean of the BP measurements taken in phase 2. Since phase 2 and 3 were repeated four times, 4 instances were generated for each subject and the final complete dataset was composed of 40 instances (4 instances * 10 subjects).

The BP7 measurement was used because it has been demonstrated that the BP drop is more frequent (observed in about 46% of patients) within 3 minutes after the standing action, in accordance with [15,16]. Since the BP measurement device reported a nominal error (NE) of ±3 mmHg, the measurement error of the ΔBP was estimated as follow:

(5) $$\text{Δ}BP_{error}\;=\;NE\;+\frac{NE}{\sqrt{n}}$$

Where *n *is the number of repeated measurements. Therefore, an error of ±4.5 mmHg was estimated on the measured ΔBP.

### Predictive model

A robust multi-linear regression [17] was used to develop a mathematical model to predict the ΔBP by using the subset *f' *of HRV features.

As stated in the introduction, the hypothesis was that the drop of BP after standing could be predicted by using the HRV measurements extracted from the ECGs registered in the 5 minutes before standing. Therefore, we modelled the ΔBP as a combination of *n *HRV features (equation 6):

(6) $$\text{Δ}BP=c_{0}+c_{1}\;f_{1}^{\prime }+...+\;c_{n}\;f_{n}^{\prime }+\varepsilon$$

where:

• c_0 _was the intercept and c_1_...n determined the contribution of the independent variable f_1_...n.

• *ε *was a random variable normally distributed.

The model was developed by using the *robustfit *function of the MATLAB version R2013a [18,19].

### Feature selection and performance evaluation

The best subset *f' *of HRV features was selected using the so-called exhaustive search method [20], investigating all the possible combinations of k out of N features (with k from 1 to n). Since the number of HRV measurements was relatively high considering the number of subjects, we limited the value of n to a maximum number of 5.

The best subset was selected as the one that minimizes the Regression Standard Error *σ*_est_, defined as follows:

(7) $$\sigma_{\text{est}}=\sqrt{\frac{\sum {\left(Y-Y^{\prime }\right)}^{2}}{N-2}}$$

where: Y was the actual value (the observed BP drop), Y' was the predicted value (the predicted BP drop), and N was the number of instances utilised to develop the model. The numerator was the sum of the squared differences between the actual scores and the predicted scores. Therefore, this error represented the average distance between the real BP drop values and the regression line. Consequently, **σ_est _**reported on how wrong the regression model was on average using the same units of the predicted value. Smaller **σ_est _**values were considered better because they indicated that the prediction was closer to the fitted line. The **σ_est _**was estimated using the leave-one-subject-out approach. Therefore, the robust regression was performed ten times, each time using 9 subjects to develop the model and one to test it. Consequently, 10 values of **σ_est _**were computed and the average of these 10 values was used to choose the best feature combination. For this combination of features, the final model was then fitted on the whole dataset (all the 10 subjects).

Moreover, the percentage of correctly predicted values (%CP), i.e. those with an error lower than the measurement error of the BP drop (4.5 mmHg), was computed as follows:

(8) $$\%CP=\frac{\sum \text{Θ}\left(\text{Δ}BP_{error}-\left|Y-\left(Y\right|,)\right)\right)}{N}$$

where Θ represents the Heaviside function [21].

Finally the rate of false positives and negatives was calculated according to the following conventions:

• "false negative"-the cases where the ΔBP was underestimated with an error above 5 mmHg (i.e., predicted ΔBP < measured ΔBP-5 mmHg)

• "false positive"-the cases where the ΔBP was overestimated with an error above 5 mmHg (i.e., predicted ΔBP > measured ΔBP + 5 mmHg)

## Results

A group of 10 volunteers, 7 women and 3 men, with a median age of 30.4 years (range 23-43 years), was enrolled in the School of Engineering of the University of Warwick. All the subjects signed informed consents and met the inclusion criteria. Each subject underwent 33 BP measurements (see Figure 1): one in phase 1; 16 in phase 2 (four times 4 measurements); and 16 in phase 3 (four times 4 measurements). For each subject 42 minutes of ECG were recorded (see Figure 1): two minutes in phase 1, 20 minutes in phase 2 (four times 5 minutes); and 20 minutes in phase 3 (four times 5 minutes). The final predictive dataset contained 40 instances as the protocol was repeated 4 times for each subject. Using these instances the best model identified was the one developed using the following 5 features: RMSSD, NN50, TINN, HF, and RPDET. The model is reported in equation 9.

$\begin{array}{c} \text{Δ}BP\;=-25.67+0.45*\left(RMSSD\right)-0.05*NN50+\\ \;\;-0.02*\left(TINN\right)-0.01*\left(HF\right)+0.3*\left(RPDET\right) \end{array}$(9)

The regression standard error calculated on the testing (leaving one out estimate), the training and the whole dataset was respectively 5.22 mmHg, 4.30 mmHg and 4.29 mmHg.

**Figure 2:**
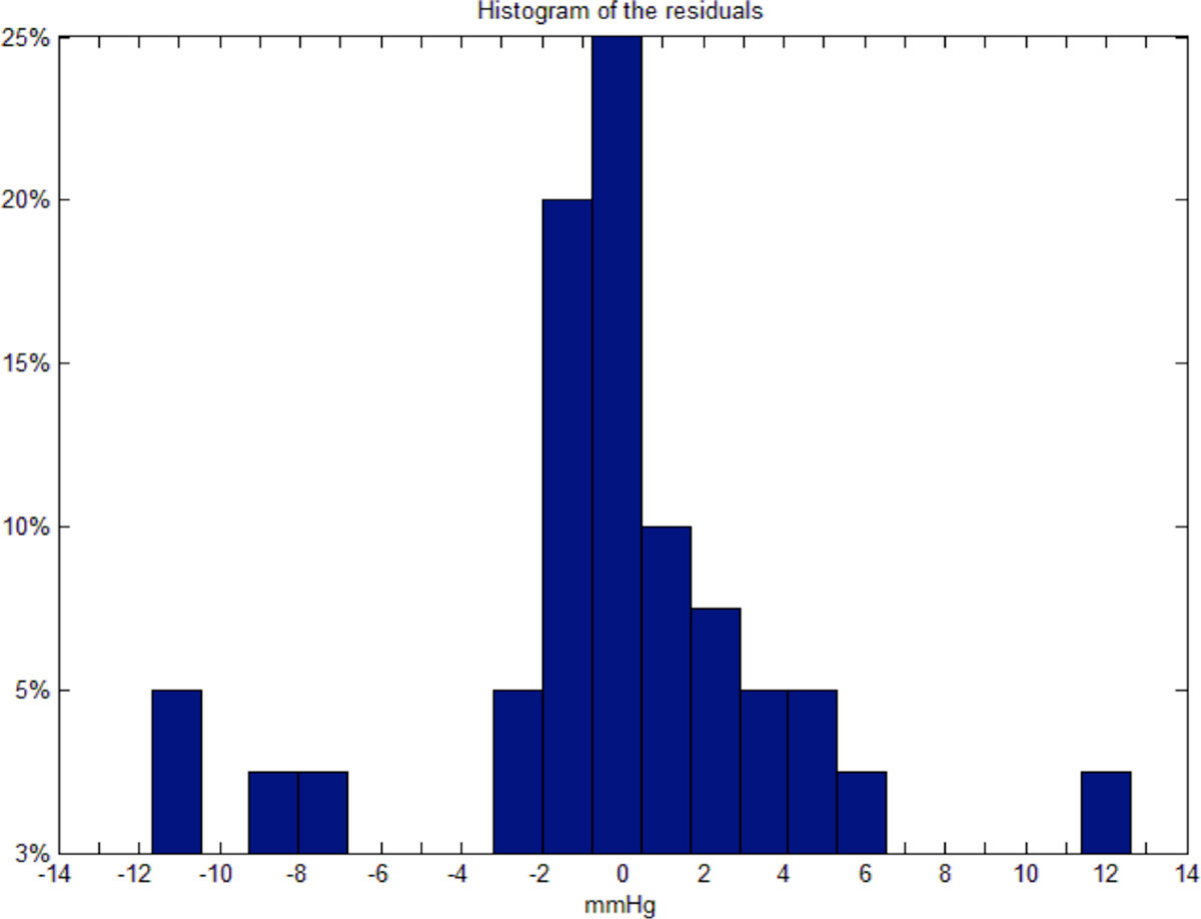
**Histogram of Residuals**. Histogram of residuals, which represents the distribution of the prediction errors. Each bar refers to the % of predictions with the error in mmHg reported below the bar. The bar in the center indicates that 25% of predictions have an error = 0; the 3 central bars indicate that 55% of predictions estimate ΔBP with an error smaller than ±2 mmHg.

The histogram of the residuals is reported in Figure 2.

**Figure 3:**
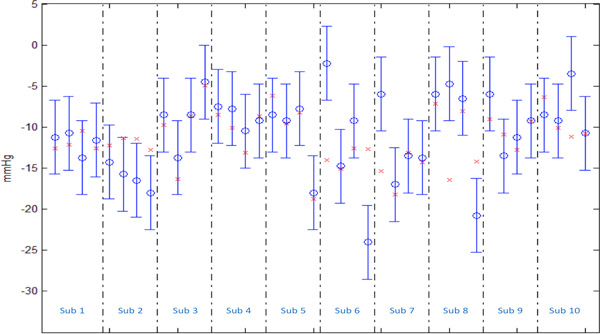
**Measured and predicted value of ΔBP**. Measured and predicted value of ΔBP after standing: each measurement shows the measured ΔBP (blue circles) with measurement error (vertical blue bars) and the predicted ΔBP (red crosses) using the model described in this paper. Each measurement was repeated 4 times per subject.

Figure 3 reports the values of ΔBP predicted by this model (red crosses) against the measured values (blue circles) and the measurement error (vertical blue error bars). The vertical dashed lines divide the four instances of each subject. The percentage of correctly predicted values (error below 4.5 mmHg) was 80%, indicating that in 32 of 40 experiments, the ΔBP was predicted with an error below the measurement error of the sphygmomanometer.

As shown in Figure 3, in 3 measurements out of 40, the measured ΔBP was underestimated with an error above 5 mmHg, resulting in a false negative rate of 7.5%: subject 2, fourth measurement, subject 6, fourth measurement, and subject 8, fourth measurement. Finally, in 4 measurements the ΔBP was overestimated, resulting in a false positive rate of 10%: subject 6, first measurement, subject 7, first measurement, subject 8, second measurement, and subject 10, third measurement.

## Discussion

This paper presents a model to predict systolic BP drop due to standing relying on HRV measurements extracted from 5 minute ECGs recorded before standing. The proposed model is based on the hypothesis that the magnitude of BP drop in the few minutes after standing is correlated with HRV features extracted from ECG recordings taken in the 5 minutes before standing. According to the mathematical model here proposed, an increased ΔBP appears to be related to:

• a reduced overall short-term HRV, as assessed by TINN and RMSSD [22].

• an increased value of HF and/or NN50, which is strongly correlated to HF [23].

• an increased determinism (as evaluated by RPDET), and, therefore, a decline of non-linear heartbeat dynamics [24].

As HF is an indicator of parasympathetic function, while the overall variability is considered a marker of both sympathetic and parasympathetic tones [25], the combined association with a reduced overall variability and increased HF suggests that an increased ΔBP could be associated with a reduction in the sympathetic tone. Mechanistically, it is assumed that the upright position gravity causes venous pooling in the lower extremities, resulting in a reduction in systemic blood pressure. This should result in a baroreflex activation, which leads to an increased sympathetic tone. Sympathetically mediated peripheral vasoconstriction and tachycardia should then occur in order to maintain the Blood Pressure value [25]. In the case of a decreased sympathetic tone, this mechanism could fail to maintain the blood pressure value and result in higher values of ΔBP.

A few previous studies have compared HRV features between subjects with and without significant standing hypotension, which is defined as a sustained reduction of systolic blood pressure of at least 20 mmHg or of diastolic blood pressure of 10 mmHg within 3 minutes of standing. In particular, Barbic et al. [26] found a decreased LF (representing a reduced sympathetic activity) in patients with Parkinson's Disease with standing hypotension. The findings of the current study are in agreement with the results of [27], which found a significant association between a depressed HRV and the risk of falling in the elderly. Specifically, this study shows that the risk of falling in elderly people with a depressed HRV is five times higher than in those without a depressed HRV (Odds Ratio 5.12, CI 95% 1.42-18.41, p < 0.01). This is explained in terms of the fact that a depressed HRV would reflect a reduced capability to react to risky situations [27].

The current study has some limitations, in particular, the small sample size and the selection of healthy volunteers, which could limit the generalizability of the results to other population subgroups, such as middle-aged and elderly individuals with comorbidities. In addition, our results should be considered as preliminary, because we have tested a novel hypothesis, which has not been explored before. Hence, these results should be corroborated by further investigations, using larger samples across a wider age range. Nevertheless, our findings are intriguing because they suggest that a reduced overall short-term HRV might represent a predictive parameter for SH, which is one of the main causes of indoor falls. Previous studies aiming to prevent falls used wearable accelerometers [28-30], pressure sensors [31], ambient sensors [32-37] or a combination of these three technologies, which had no other direct benefit for later life problems. In contrast, the model proposed in this study is based on features extracted from HRV, which has been associated with a number of other health outcomes. Therefore, the clinical implications of these findings are potentially relevant, since these parameters are based on simple and non-invasive measurements. Moreover, recent systematic reviews, investigating the independent capability of different technologies to prevent falls [38-42], have highlighted their limitations, in particular, the rate of false alarms (16%), which is too high to maintain the full attention of the nursing staff[38]. The model of the current study is estimated to achieve a lower false positive rate (10%) and could be enhanced by the addition of other sensor information, for example, accelerometric signals and breath rates, which are already acquired by the adopted wearable device.

## Conclusions and future works

This study suggests that the lowering of blood pressure in the few minutes after standing can be predicted by monitoring HRV features recorded in the 5 minutes before standing. Particularly, more significant blood pressure decreases were observed in subjects presenting a depressed and less chaotic HRV pattern. This could reflect a transient depressed autonomic response causing a slower recovery of homeostasis and adjustment of blood pressure.

As a future work we will perform a large-scale experimental phase in which we will recruit more subjects across a wider age range to investigate the generalizability of the results obtained in this preliminary study. Moreover, further studies will investigate the influence of other factors, e.g., time of day, previous medications, and cardiovascular disease.

In this way we can refine the developed mathematical model, if necessary, in order to predict falls by detecting the cardiovascular and autonomous nervous system states (CVS/ANS). Additionally, the model will be implemented and embedded in a real-time mobile monitoring system in order to inform the patient and, in the case of a hospitalised patient, the medical staff about the high possibility of having a significant drop in blood pressure two minutes before this might happen. In any case where the model predicts a fall, the system can recommend to the patient not to perform any fast standing action alone.

## List of abbreviations

ΔBP: blood pressure lowering due to standing

ECG: ElectroCardioGram

HRV: Heart Rate Variability

BP, Blood Pressure;

SH, Standing Hypotension;

SYS, Systolic Blood Pressure;

DIA, Diastolic Blood Pressure;

HDFP, Hypertension Detection and Following-up Program;

NHS, National Health Service;

ANS, Autonomous Nervous System;

OR, Odds Ratio;

CI, Confidence Interval;

BSREC, Biomedical and Scientific Research Ethics Committee;

%CP, Percent correct prediction;

mmHg, millimeter of mercury;

LF, low frequency;

HF, High frequency.

## Competing interests

The authors declare that they have no competing interests.

## Authors' contributions

GS refined the study protocol, collected the data, processed the signals, and drafted the first version of manuscript. PM contributed to the predictive model development, and to the writing of the manuscript. SS contributed to the study design, to the preparation of the ethical approval submission, the clinical interpretation of the findings and to writing of the paper. PDP contributed to the writing of the paper. LP conceived the study, prepared the ethical approval, coordinated the study execution, developed the predictive model and contributed to the writing of the paper. All the authors read and approved the final version of this paper
